# Cardiovascular Autonomic Neuropathy and Glucose Variability in Patients With Type 1 Diabetes: Is There an Association?

**DOI:** 10.3389/fendo.2018.00174

**Published:** 2018-04-19

**Authors:** Szabolcs Nyiraty, Fruzsina Pesei, Andrea Orosz, Sara Coluzzi, Orsolya Eszter Vági, Csaba Lengyel, György Ábrahám, Simona Frontoni, Peter Kempler, Tamás Várkonyi

**Affiliations:** ^1^First Department of Medicine, University of Szeged, Szeged, Hungary; ^2^Department of Pharmacology and Pharmacotherapy, University of Szeged, Szeged, Hungary; ^3^Department of Systems Medicine, University of Rome Tor Vergata, Rome, Italy; ^4^Unit of Endocrinology, Diabetes and Metabolism, S. Giovanni Calibita Fatebenefratelli Hospital, Rome, Italy; ^5^First Department of Medicine, Semmelweis University, Budapest, Hungary

**Keywords:** autonomic neuropathy, glucose variability, continuous glucose monitoring, type 1 diabetes, cardiovascular reflex tests

## Abstract

**Introduction:**

The oxidative stress associated with glucose variability might be responsible for neuronal damage while autonomic neuropathy (AN) has a detrimental effect on metabolism. The aim of the study was to find relationship between AN and GV in type 1 diabetic patients and to identify further factors that affect GV.

**Patients and methods:**

Twenty type 1 diabetic patients were involved (age: 39.5 ± 3.4 years, duration of diabetes: 17.5 ± 2.5 years; HbA1c: 8.1 ± 0.2%, mean ± SE). AN was assessed by the cardiovascular reflex tests. The interstitial glucose levels were determined following insertion of a subcutaneous electrode during the continuous glucose monitoring (CGM) method on six consecutive days. GV was characterized by calculation of four parameters.

**Results:**

SD of interstitial glucose values correlated positively with the overall AN score and the degree of the orthostatic reduction of systolic blood pressure (AN-score-SD ρ = 0.47, *p* < 0.05; orthostasis-SD: ρ = 0.51, *p* < 0.05). Mean absolute glucose (MAG) correlated with three parameters of AN (AN-score-MAG: ρ = 0.62, *p* < 0.01; 30/15 ratio-MAG: ρ = −0.50, *p* < 0.05; orthostasis-MAG: ρ = 0.59, *p* < 0.01). The HbA1c also correlated with two parameters of GV (HbA1c-continuous overlapping net glycemic action: ρ = 0.56, *p* < 0.05; HbA1c-MAG: ρ = 0.45, *p* < 0.05). The frequency of hypoglycemia did not exhibit any correlation with measures of GV.

**Conclusion:**

Severity of glucose variability but not overall glucose load correlates with both parasympathetic and sympathetic dysfunctions in type 1 diabetes. Higher HbA1c is associated with more severe glucose variability. The observed correlation between increased glucose variability and the severity of AN necessitates the further exploration of this relationship.

## Introduction

Large prospective trials provided clear evidence two decades ago that long-term hyperglycemia due to less intensive treatment is associated with micro- and macrovascular complications in patients with diabetes (1, 2). Later the detrimental role of recurrent hypoglycemic episodes and acute as well as chronic hyperglycemia was also proven in the development of severe disorders of several important organ systems (3, 4). Fasting and postprandial glucose as well as HbA1c are generally used in clinical studies and the everyday practice to express the glycemic control of diabetic patients. However, the value of all three parameters is limited as fluctuations of glucose are not characterized by them (5). Thus, the concept of glucose variability (GV) was introduced to describe the variations of glucose levels (6). The variability of blood or interstitial glucose as well as HbA1c reflects the level of deviations from the mean value of these parameters (7). Keeping the strict balance of carbohydrate metabolism is a real challenge in a diabetic patient as several factors may influence the actual glucose values such as age, cognitive impairment, or liver and kidney failure (8). Moreover, glycemic stability is influenced by antidiabetic therapy, diet, and body composition (9). Reduced beta-cell function is one of the most important risk factors of GV and an inverse relationship between residual C-peptide levels and glucose variability has been shown in type 1 diabetic patients (10). Beta-cell dysfunction might be associated with glycemic instability in type 2 diabetic patients as well (11). In type 1 diabetic patients, hyperglycemia is primarily related to the loss of endogenous insulin secretion, while the impaired glucagon response to hypoglycemia explains the susceptibility to abnormally low glucose values (12). These observations suggest the hypothesis of a more pronounced GV in type 1 than in type 2 diabetic subjects. However, most of the previous studies about the detrimental effects of GV were conducted on type 2 diabetic patients, and thus the possible differences in causes and consequences of GV between type 1 and type 2 diabetic patients have not been clearly analyzed up to now. Exploration of the factors responsible for this metabolic situation in type 1 diabetes is an important aim of a clinical research. The potential role of autonomic neuropathy (AN) in the development of higher GV should be studied as well. There is a considerable interplay between diabetic neuropathy and GV. On the one hand, the potential role of GV in the development of neuronal damage is discussed (13). On the other hand, manifestations of AN and peripheral neuropathy may lead to metabolic imbalance (14). In the present study, we aimed to assess a potential connection between GV and cardiovascular AN among patients with type 1 diabetes. We also investigated further possible pathogenetic factors of GV including HbA1c, body mass index (BMI), gender, age, daily insulin dose, diabetes duration, and frequency of hypoglycemia.

## Materials and Methods

### Patients

Twenty type 1 diabetic patients with diabetes were involved in the study (age: 39.5 ± 3.4 years, duration of diabetes: 17.5 ± 3.4 years; mean ± SE). They were non-obese (BMI: 22.3 ± 0.8 kg/m^2^) and their mean HbA1c was 8.1 ± 0.7%. All patients applied multiple daily injections of analog insulins, their mean daily insulin dose was 42.8 ± 2.9 U. Further anthropometric and clinical parameters of the patients can be found at Table S1 in Supplementary Material. Patients with atrial fibrillation, acute infection, thyroid disease, or chronic alcohol consumption were excluded from the study. The study was approved by the Regional Scientific and Research Ethical Committee of the Albert Szent-Györgyi Health Center at University of Szeged. All subjects have given written informed consent of the study.

### Assessment of AN

Cardiovascular consequences of AN were detected to characterize the presence and severity of the neuronal dysfunction. The five standard cardiovascular reflex tests (CRT) were applied (15). These measurements provide a non-invasive, clinically relevant, reproducible, and standardized gold-standard determination of autonomic function (16). Three of these tests record the change of heart rate during specific maneuvers, while other two tests were designed to evaluate blood pressure changes (17). Most of the tests aiming to detect changes in heart rate are used primarily (but not exclusively) for the assessment of parasympathetic innervation, while the blood pressure responses predominantly reflect the impairment of sympathetic functions. The heart rate changes were analyzed during deep breathing, in positions of lying and standing up (30/15 ratio), and during and after of Valsalva maneuver. Systolic blood pressure was determined in response from lying to standing up, and diastolic pressure change was measured during a sustained handgrip. The results of each CRT were correlated with the calculated parameters of GV. Finally each CRT was scored as 0 (normal), 1 (borderline), or 2 (abnormal), and by this method an autonomic score (0–10) was calculated to express the overall severity of AN. Age-corrected normal reference values were applied based on the definition of Ewing and the recommendations of the Toronto Neuropathy Expert Group (17, 18). The tests were performed in postprandial state, after breakfast. In case of actual hypo- or hyperglycemia, the tests were not done.

### Determination of GV

Glucose variability was calculated from the results of a continuous glucose monitoring (CGM) system (Ipro 2 with Enlite sensor, Medtronic Minimed Inc.). During this procedure, a tiny flexible electrode was placed under the skin. The electrode continuously measured the glucose levels of the interstitial fluid. 288 glucose readings were detected per 24 h and the total period of continuous measurement was 6 days. The measured glucose values became available following the download of the detected data. SD, mean amplitude of glycemic excursions (MAGE), continuous overlapping net glycemic action (CONGA), and mean absolute glucose (MAG) of interstitial fluid were calculated to characterize GV (19).

### Analysis of Possible Pathogenetic Factors Responsible for Glucose Variability

To identify potential factors impacting on GV, HbA1c, BMI, age, diabetes duration, and daily insulin dose of patients were explored. Different categories of hypoglycemia were also characterized. Measured hypoglycemia was defined when blood glucose was below 3.9 mmol/L detected by continuous glucose monitoring system (CGMS). Severe hypoglycemia was categorized if serious cognitive impairment requiring assistance from another person associated with blood glucose lower than 3.9 mmol/L occurred. Hypoglycemia unawareness was established if a measured hypoglycemia was not recognized by the patient.

Statistical analyses between different parameters of glucose variability and the patients’ characteristics were performed with the Spearman correlation test and multiple regression analysis. Statistical significance was defined by *p* < 0.05. The statistical analyses were performed using the SigmaStat 4.0 Systat Software package.

## Results

**Figure 1:**
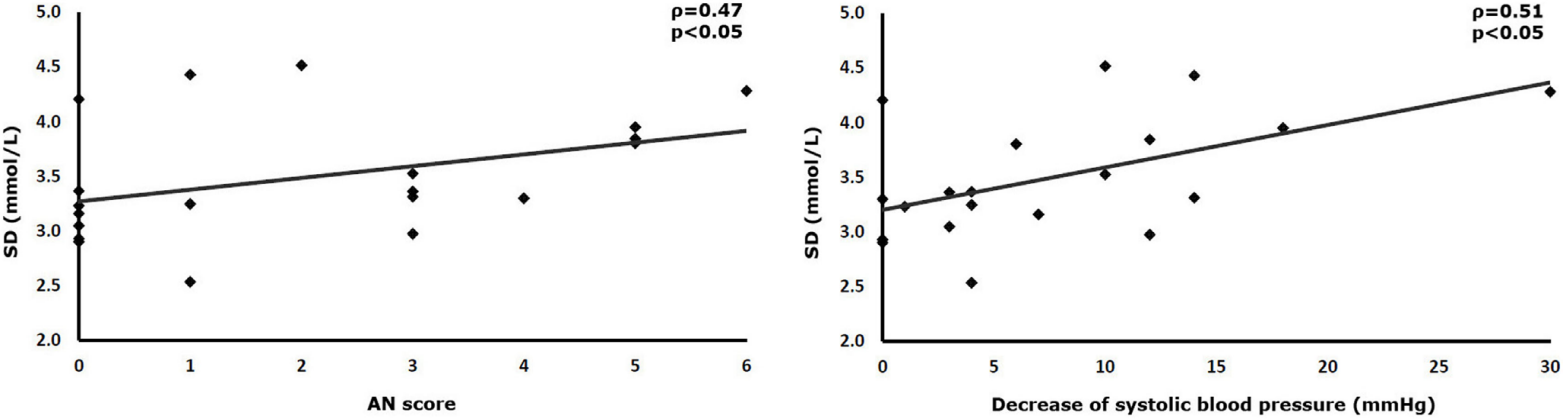
Correlations between SD of continuously measured interstitial glucose levels and cardiovascular reflex tests.

**Figure 2:**
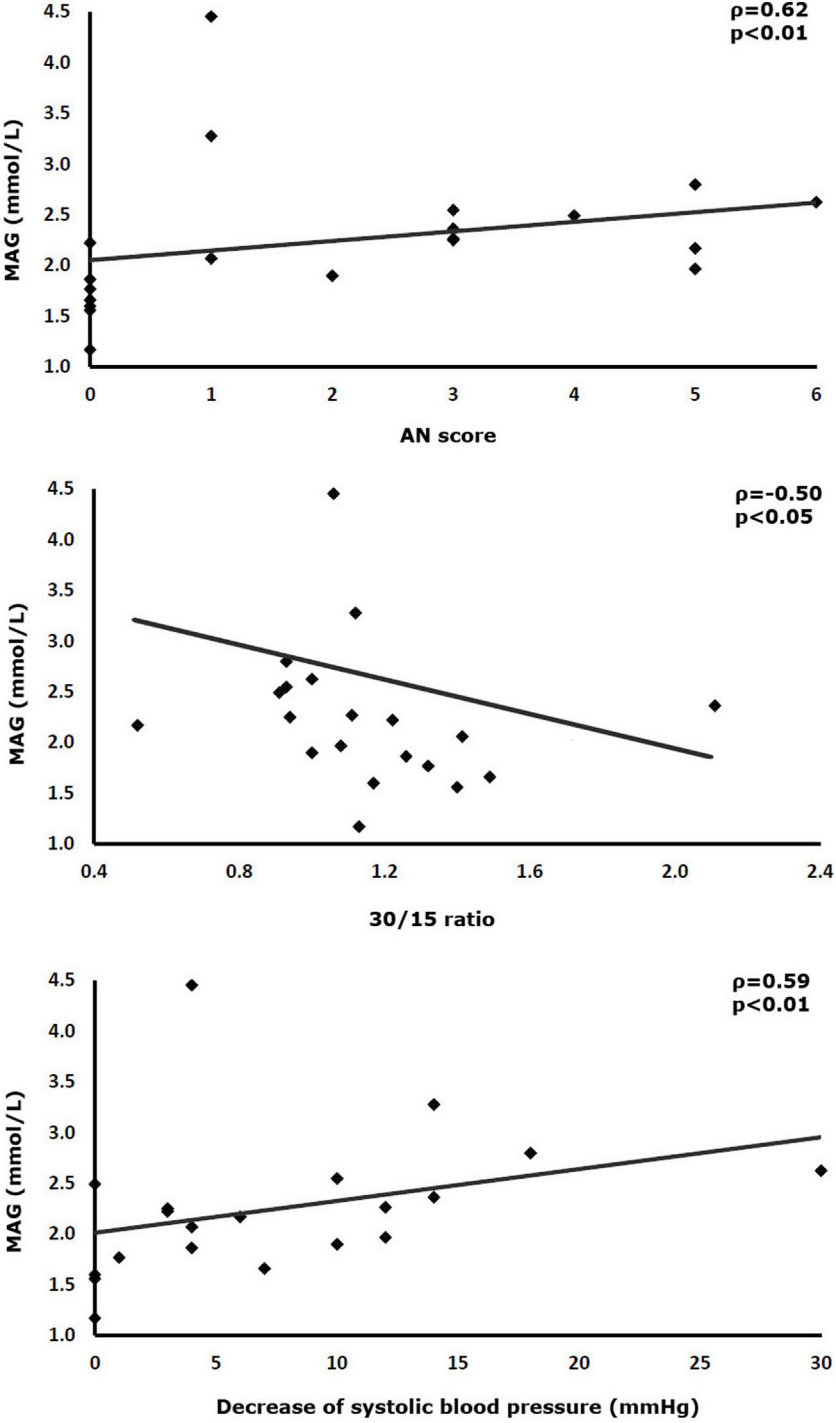
Correlations between the mean absolute glucose (MAG) of continuously measured interstitial glucose levels and cardiovascular reflex tests.

**Figure 3:**
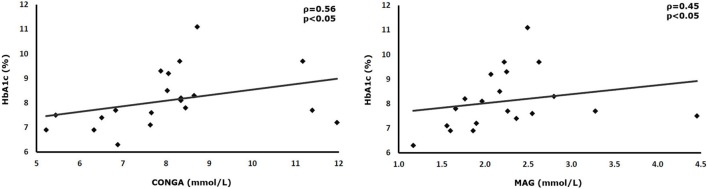
Correlations between glucose variability parameters and HBA1c.

The mean values of the measured CRT and variability parameters in type 1 diabetic patients are listed in Table 1. The CRT mean values of the patient group reflected a moderate autonomic impairment, while all measured mean GV parameters of the patients were higher than the previously published reference values in healthy subjects (19). As a next step of the analysis, the patients were divided into two groups: patients with AN scores 0–1 (*n* = 10) and patients with AN scores 2–10 (*n* = 10). The GV parameters were compared and no significant difference was proven between the groups with a tendency of higher GV parameters in the AN group (CONGA: 7.6 ± 0.55 vs 8.5 ± 0.56 mmol/L, *p* = 0.235; SD: 3.3 ± 0.15 vs 3.67 ± 0.18 mmol/L, *p* = 0.129, MAGE: 5.9 ± 0.4 vs 6.2 ± 0.16 mmol/L, *p* = 0.678; MAG: 2.16 ± 0.3 vs 2.33 ± 0.09 mmol/L, *p* = 0.06; patients without AN vs patients with AN, mean ± SE). The further analyses were done on the whole group (*n* = 20). The AN scores calculated from the CRT-s expressing the overall severity of cardiovascular AN correlated positively with the SD of continuously measured interstitial glucose levels (ρ = 0.47, *p* < 0.05; Figure 1) thus showing that higher glucose variability expressed with SD was associated with more severe cardiovascular AN in this group of type 1 diabetic patients. The statistical analysis revealed a further positive correlation between SD of the continuously measured glucose values and the systolic blood pressure response to standing (ρ = 0.51, *p* < 0.05; Figure 1). This observation reflects pronounced systolic fall of blood pressure due to sympathetic AN in the presence of GV characterized by higher SD. The relationship between GV and AN was further strengthened by the fact that MAG, a marker of GV correlated positively with the AN scores of the patients (ρ = 0.62, *p* < 0.01; Figure 2). Higher MAG values were associated with significantly lower results of the 30/15 ratio (heart rate response to standing). The negative correlation coefficient (ρ = −0.5, *p* < 0.05) reflects impaired cardiovascular autonomic function among patients with more severe GV (Figure 2). Similarly to SD, MAG also correlated positively with the degree of orthostatic systolic blood pressure fall supporting the association between GV and sympathetic dysfunction (ρ = 0.59, *p* < 0.01; Figure 2). When AN scores were also calculated after the exclusion of the handgrip tests, similarly, significant correlations were found between SD, MAG, and AN scores (SD-AN score: ρ = 0.62, *p* < 0.01, MAG-AN score: ρ = 0.51, *p* < 0.05). When the correlations were adjusted for HbA1c, age, and duration of diabetes at a multivariate analysis the relationship between SD and the systolic blood pressure response to standing remained significant (*r* = 0.49, *p* < 0.05). Higher HbA1c levels were associated with increased GV as measured by CONGA (Figure 3) or MAG (Figure 3). This observation was proven by the positive statistical correlation between HbA1c and CONGA (ρ = 0.56, *p* < 0.05) and MAG (ρ = 0.45, *p* < 0.05). No statistical correlations were found between age, duration of diabetes, daily insulin dose, or BMI of type 1 diabetic patients and various parameters of their GV. Finally, no associations were proven between glucose levels below 3.9 mmol/L and markers of GV or AN. Similarly, lack of correlation was observed between the number of severe hypoglycemic episodes or hypoglycemia unawareness and the GV parameters or the severity of AN (Table S2 in Supplementary Material).

**Table 1:** Results of cardiovascular reflex tests and glucose variability parameters in type 1 diabetic patients.

Autonomic neuropathy (AN) test	Type 1 diabetic patients (mean ± SE)
Heart rate response to deep breathing	23.9 ± 2.6 (beats/min)
30/15 ratio	1.15 ± 0.07
Valsalva ratio	1.72 ± 0.13
Diastolic blood pressure response to handgrip	16.2 ± 2.7 (mm Hg)
Systolic blood pressure response to standing up	7.6 ± 1.4 (mm Hg)
AN score	2.1 ± 0.4
Patients without AN	10/20
Patients with early AN	5/20
Patients with severe AN	5/20
**GV parameter**	
Continuous overlapping net glycemic action	8.09 ± 0.4 mmol/L
SD	3.49 ± 0.1 mmol/L
Mean amplitude of glycemic excursions	6.12 ± 0.2 mmol/L
Mean absolute glucose	2.25 ± 0.2 mmol/L

## Discussion

Our data show for the first time an association between cardiovascular AN and higher GV in type 1 diabetic patients. The positive and negative significant statistical associations found in our study indicate increasing values of MAG and SD in the presence of more severe AN reflected by two CRT-s or the AN score. We have also found higher HbA1c in the presence of increased glucose variability, while patterns of hypoglycemia were not associated with AN or glucose variability. These results supplement the previously published observations in the literature.

Altered motor and sensory axonal functions were found in a study in patients with type 1 diabetes in the presence of high MAGE, an important marker of GV acquired from continuous interstitial glucose monitoring (20). In another study, type 1 diabetic patients with painful neuropathy had a higher mean glucose, a greater M-value, and more glycemic excursions compared with the painless group (21).

In DCCT, conducted on type 1 diabetic patients, GV calculated from the 7-point daily glucose profiles was not associated with the risk of development of retinopathy, nephropathy, or cardiovascular AN. The discrepant findings in DCCT with our study might be explained by different methods: the 7-point daily glucose profile is less sufficient to express GV than CGM applied in our study. Moreover, 30/15 ratio from the CRT-s was not performed in DCCT while we used it (22). Although GV often occurs in type 1 diabetic patients, the possible associations of this metabolic imbalance and neuropathy were more frequently analyzed in type 2 diabetic patients (23, 24). Glucose variability was identified as a risk factor of macrovascular complications as well as cardiovascular and malignant diseases but only in type 2 diabetic patients (25, 26). The connection between AN and hyper/or hypoglycemia is well documented. Autonomic nerve dysfunction is related to decreased hypoglycemia awareness leading to late realization and non-efficient management of abnormally low glucose (27). Impaired counter-regulatory response of epinephrine, norepinephrine to hypoglycemia was found in AN explaining the clear association between the occurrence of severe hypoglycemia and advanced cardiovascular AN (28, 29). Finally, hypoglycemia-associated autonomic failure has been described (30). On the other hand, parasympathetic autonomic dysfunction has been shown to be associated with postprandial hyperglycemia among newly diagnosed type 2 diabetic patients (31).

Slower gastric emptying mainly as a consequence of a parasympathetic involvement develops during the progression of diabetic neuronal damage (32). Gastroparesis in AN is considered as the underlying mechanism in patients with unexplained periods of hypoglycemia followed by hyperglycemia (33, 34). Abnormal gastrointestinal peptide release due to autonomic dysfunction (including pancreatic polypeptide, motilin) causes additional motility and secretory dysfunctions that result in abnormal carbohydrate absorption (35).

In our study, several parameters of GV acquired from continuous interstitial glucose measurement associated with results of CRT-s and HbA1c of type 1 diabetic patients with disease. Continuous interstitial glucose detection provides a more detailed glucose time series than the self-monitored capillary glucose sampling or the variability of HbA1c. This method ensures the calculation of at least 10 parameters that describe glycemic stability of diabetic patients (19). The mean values of GV parameters reflected serious glycemic instability in this group of patients with type 1 diabetes (Table 1). SD as the most widely applied parameter that shows a linear relationship with mean glucose (9) correlated with the overall severity of cardiovascular AN and the systolic blood pressure response to standing. The SD of interstitial glucose values is higher in the presence of more severe AN and if blood pressure fall is more pronounced to standing. The orthostatic hypotension is a characteristic late symptom of advanced neuropathy and reflects a sympathetic dysfunction. This sympathetic impairment is frequently associated with altered norepinephrine levels which might explain an abnormal counter-regulatory response to hypoglycemia. The other glucose variability marker, MAG, reflected more unstable glucose in our study, if the cumulative autonomic score was higher and two reflex tests were more abnormal. MAG is a summation of all absolute changes in glucose, divided by the time elapsed during the measurements. One of the two reflex tests that correlated with MAG was a ratio of the heart rate responses to standing reflecting mainly the parasympathetic function, while the other, the orthostatic systolic blood pressure to standing supplied information on sympathetic function. Thus, parasympathetic and sympathetic functions are both altered if GV is enhanced. The statistical correlation between the measured glucose variability and cardiovascular autonomic functions support a final conclusion that there might be a causal relationship between glycemic instability and AN. The original correlations between AN score and GV indices were reproduced, when handgrip tests were excluded from the calculation of the AN scores of the patients. The low sensitivity and specificity of the handgrip test in the diagnosis of cardiovascular AN and its high dependency on hypertensive status and baseline diastolic BP were proven before (36). The similar significant correlations without handgrip tests support the hypothesis that handgrip has a low value in the measurement of AN and strengthens the observed correlations between autonomic function and GV. The relationship between AN and GV was clearly proven, although the mean severity of AN was moderate of the patients, while GV was severe (Table 1).

As an interesting finding, we detected two correlations between markers of GV and HbA1c. The general approach of the literature to the possible relationship of GV and HbA1c is that these parameters reflect different patterns of carbohydrate metabolism: parameters of GV calculated from CGMS describe both hypo- and hyperglycemic episodes for a short-term period, while HbA1c reflects mean blood glucose and primarily driven by the extent of hyperglycemia (37). One explanation of our finding is that the patients in this study might have spent more time in hyperglycemia than hypoglycemia if their variability markers were high. The hypoglycemic episodes were frequently followed by hyperglycemia and these intervals might have been added to the “purely” hyperglycemic episodes. The frequency or severity of the realized or measured hypoglycemic episodes in this study did not correlate with GV markers pointing also to the longer hyperglycemic intervals in our patients. The mean value of the HbA1c of the patients was over the target (8.1%) supporting the long-standing hyperglycemia. Analyzing the variability markers separately, MAG is relatively weakly associated with hypoglycemia and reflects more hyperglycemia. On the other hand, calculation of MAG includes a timing component and is not a purely amplitude describing parameter that might explain the parallel change with HbA1c (38). The other parameter, CONGA is calculated from the difference between a current and earlier glucose measurements and expresses the SD of these differences reflecting definitely the timing of variability. These markers incorporating a time-dependent description of GV might have parallel kinetics with the change of HbA1c (39). The associations between HbA1c and GV as well as GV and AN allow conclusions that the higher HbA1c is responsible for AN and AN leads to GV but it also could be assumed that GV manifests in higher HbA1c that results AN.

We did not find significant correlation between number of any hypoglycemia, severe hypoglycemic episodes, or hypoglycemia unawareness and the severity of AN in our patients. In the EURODIAB IDDM Complications Study, only in case of a combined autonomic deficit in heart rate and blood pressure responses to standing was associated with a modest increase in the risk of severe hypoglycemia. Most of patients in our study had only a moderate AN. Moreover, in adults with type 1 diabetes in another study no relationship was found between hypoglycemia unawareness and autonomic dysfunction similarly with our observations (29, 40). A study on type 1 diabetic patients, however, had conflicting data with our findings in which autonomic function characterized with heart rate variability correlated with low blood glucose index and area under the curve for hypoglycemia of CGM measures (41). Thus, application of different methods from ours revealed correlation between autonomic function and hypoglycemia that necessitates further explorations about this relationship.

Several factors may impact GV, particularly longer duration of diabetes is associated with higher GV. However, when the correlations were adjusted for HbA1c, age, and diabetes duration at a multivariate analysis, the relationship between SD and the systolic blood pressure response to standing remained significant (*r* = 0.49, *p* < 0.05). Although the moderate sample size may explain why only the correlation between SD and orthostatic hypotension remained significant after adjustment for confounding variables, it is noteworthy to stress the concept that in the presence of advanced AN a severe variability of glucose is observed in type 1 diabetic patients. Orthostatic hypotension is a characteristic sign of the late progressive stages of AN (17) and primarily refers to the impairment of the sympathetic autonomic function. Orthostatic blood pressure response was among the two tests associated with a high risk of severe hypoglycemia in the EURODIAB IDDM Complications Study (29). The increased risk of hypoglycemia leads to pronounced GV in patients with impaired sympathetic function and is explained by the impaired counter-regulatory response to abnormally low glucose levels. The relatively low number of patients might be considered as a limitation of our study. Nevertheless, a similar or even smaller number of diabetic patients were recruited in other studies on the same field (27, 28, 42, 43). It should be added that according to the protocol of our study, AN and CGMS assessments were performed only in those cases in which both were indicated as a part of a medical management. The lack of the assessment of the symptoms of AN are among the limitations although these would predict the possible presence of autonomic dysfunction. A further limitation is that not all but two of four GV parameters correlated with the AN tests. One of these parameters is the most frequently applied standard GV marker, the SD by which our data are comparable with further studies.

## Conclusion

It can be concluded from our study that in type 1 diabetes, increased GV is in a close relationship with advanced AN and might be manifested in higher HbA1c. Due to our study design, it is not possible to differentiate whether glucose variability induces neuropathy or the glycemic instability is a consequence of neuropathy. The nature of these relationships should be explored in further studies.

## Ethics Statement

This study was carried out in accordance with the recommendations of The Regional Human Biomedical Research Ethics Committee of University of Szeged with written informed consent from all subjects. All subjects gave written informed consent in accordance with the Declaration of Helsinki. The protocol was approved by the Regional Human Biomedical Research Ethics Committee of University of Szeged.

## Author Contributions

SN, FP, AO, SC, OV, GÁ, CL, SF, PK, and TV had substantial contributions to the conception of the work and design of the paper, read and approved the final manuscript. SN, FP, AO, SC, and TV contributed to the measurements and analyses of data. GÁ, SF, PK, and TV drafted the paper or revised it critically for important intellectual content.

## Conflict of Interest Statement

The authors declare that the research was conducted in the absence of any commercial or financial relationships that could be construed as a potential conflict of interest. The reviewer VS declared a shared affiliation, with no collaboration, with one of the authors SF to the handling Editor.

## Abbreviations

AN, autonomic neuropathy; BMI, body mass index; CGM, continuous glucose monitoring; CGMS, continuous glucose monitoring system; CONGA, continuous overlapping net glycemic action; CRT, cardiovascular reflex test; GV, glucose variability; MAG, mean absolute glucose; MAGE, mean amplitude of glycemic excursions.
