# Supplementary material for: Cardiovascular Autonomic Neuropathy and Glucose Variability in Patients With Type 1 Diabetes: Is There an Association?
Source: Front Endocrinol (Lausanne). 2018 Apr 19;9:174. doi: 10.3389/fendo.2018.00174 (PMC5916962; doi:10.3389/fendo.2018.00174)
Supplement: Supplementary file 1 [file data_sheet_1.docx]

**Supplementary material**

**Table S1 Anthropometric and clinical parameters of subjects with type 1 diabetes (mean±SE)**

| **variable** | **type 1 diabetic patients (n=20)** |
| --- | --- |
| **age (years)** | **39.5±3.4** |
| **gender (male/female)** | **6/14** |
| **HbA1c ( %)** | **8.1±0.7** |
| **duration of diabetes (years)** | **17.5±3.4** |
| **BMI (kg/m^2^)** | **22.3±0.8** |
| **GFR (mL/min)** | **75.3±2.4** |
| **daily insulin dose (Units/day)** | **42.8±2.9** |
| **systolic blood pressure (mm Hg)** | **114±2.1** |
| **diastolic blood pressure (mm Hg)** | **74.4±1.8** |
| **patients with retinopathy** | **5** |
| **patients with microalbuminuria** | **1** |
| **patients with hypertension** | **5** |
| **patients with CV disease** | **0** |

**Table S2 Results of correlations between clinical variables and GV, hypoglycemia or CAN**

| **correlated parameters** | **correlation coefficient** | **p value** |
| --- | --- | --- |
| **diabetes duration- CONGA** | **-0.063** | **0.787** |
| **diabetes duration – SD** | **0.202** | **0.385** |
| **diabetes duration – MAGE** | **0.151** | **0.521** |
| **diabetes duration- MAG** | **-0.171** | **0.464** |
| **BMI – CONGA** | **-0.227** | **0.330** |
| **BMI- SD** | **-0.197** | **0.399** |
| **BMI-MAGE** | **0.081** | **0.729** |
| **BMI-MAG** | **0.103** | **0.663** |
| **age – CONGA** | **-0.009** | **0.962** |
| **age – SD** | **0.424** | **0.061** |
| **age – MAGE** | **0.245** | **0.292** |
| **age-MAG** | **-0.153** | **0.513** |
| **HbA1c – SD** | **0.123** | **0.599** |
| **HbA1c- MAGE** | **-0.224** | **0.337** |
| **AN score – CONGA** | **0.340** | **0.140** |
| **AN score – MAGE** | **0.214** | **0.357** |
| **heart rate response to breathing – CONGA** | **-0.119** | **0.612** |
| **heart rate response to breathing – SD** | **-0.333** | **0.147** |
| **heart rate response to breathing – MAGE** | **-0.092** | **0.695** |
| **heart rate response to breathing – MAG** | **-0.003** | **0.987** |
| **Valsalva ratio – CONGA** | **-0.046** | **0.841** |
| **Valsalva ratio –SD** | **-0.278** | **0.230** |
| **Valsalva ratio –MAGE** | **-0.118** | **0.612** |
| **Valsalva ratio –MAG** | **-0.346** | **0.132** |
| **30/15 ratio – CONGA** | **-0.284** | **0.220** |
| **30/15 ratio –SD** | **-0.311** | **0.178** |
| **30/15 ratio – MAGE** | **-0.265** | **0.255** |
| **systolic blood pressure decrease - CONGA** | **0.358** | **0.119** |
| **systolic blood pressure decrease – MAGE** | **0.154** | **0.508** |
| **AN score- number of hypogycemic events** | **-0,069** | **0,767** |
| **AN score - number of severe hypogycemic events** | **0.143** | **0.542** |
| **AN score - ratio of percepted hypoglycemias** | **0.258** | **0.269** |
